# Correlation between toxicity and dosimetric parameters for adjuvant intensity modulated radiation therapy of breast cancer: a prospective study

**DOI:** 10.1038/s41598-021-83159-3

**Published:** 2021-02-11

**Authors:** David Pasquier, Benoit Bataille, Florence Le Tinier, Raoudha Bennadji, Hélène Langin, Alexandre Escande, Emmanuelle Tresch, Franck Darloy, Damien Carlier, Frederik Crop, Eric Lartigau

**Affiliations:** 1 Academic Department of Radiation Oncology, Centre Oscar Lambret, 3 rue Frédéric Combemale, 59000 Lille, France; 2 CRIStAL UMR CNRS 9189, Lille University, Avenue Carl Gauss, 59650 Villeneuve-d’Ascq, France; 3 Methodology and Biostatistics Unit, Centre Oscar Lambret, 3 rue Frédéric Combemale, 59000 Lille, France; 4 Service de Radiothérapie, Clinique du Pont Saint-Vaast, Centre Léonard de Vinci, 2 rue du Pont Saint-Vaast, 59500 Douai, France; 5 Medical Physics Department, Centre Oscar Lambret, 3 rue Frédéric Combemale, 59000 Lille, France

**Keywords:** Oncology, Cancer

## Abstract

ORCID: 0000–0001-6019–7309**. **In the treatment of breast cancer, intensity-modulated radiation therapy (IMRT) reportedly reduces the high-dose irradiation of at-risk organs and decreases the frequency of adverse events (AEs). Comparisons with conventional radiotherapy have shown that IMRT is associated with lower frequencies of acute and late-onset AEs. Here, we extended a prospective, observational, single-center study of the safety of IMRT to a second investigating center. Patients scheduled for adjuvant IMRT after partial or total mastectomy were given a dose of 50 Gy (25 fractions of 2 Gy over 5 weeks), with a simultaneous integrated boost in patients having undergone conservative surgery. 300 patients were included in the study, and 288 were analyzed. The median follow-up period was 2.1 years. The 2-year disease-free survival rate [95% CI] was 93.4% [89.2–96.0%]. Most AEs were mild. The most common AEs were skin-related—mainly radiodermatitis [in 266 patients (92.4%)] and hyperpigmentation (in 178 (61.8%)). 35% and 6% of the patients presented with grade 2 acute skin and esophageal toxicity, respectively. Only 4 patients presented with a grade 3 event (radiodermatitis). Smoking (odds ratio) [95% CI] = 2.10 [1.14–3.87]; p = 0.017), no prior chemotherapy (0.52 [0.27–0.98]; p = 0.044), and D98% for subclavicular skin (1.030 [1.001–1.061]; p = 0.045) were associated with grade ≥ 2 acute AEs. In a univariate analysis, the mean dose, (p < 0.0001), D2% (p < 0.0001), D50% (p = 0.037), D95% (p = 0.0005), D98% (p = 0.0007), V30Gy (p < 0.0001), and V45Gy (p = 0.0001) were significantly associated with grade ≥ 1 acute esophageal AEs. In a multivariate analysis, D95% for the skin (p < 0.001), D98% for the subclavicular skin and low D95% for the internal mammary lymph nodes were associated with grade ≥ 1 medium-term AEs. The safety profile of adjuvant IMRT after partial or total mastectomy is influenced by dosimetric parameters.

*Trial registration*: ClinicalTrials.gov NCT02281149.

## Introduction

Radiotherapy is recommended as an adjuvant treatment for breast cancer after breast-conserving surgery or after mastectomy in patients with node-positive disease; it is associated with significant reductions in the risk of recurrence and long-term cancer mortality^1,2^. The current standard of care is three-dimensional conformational radiotherapy (3D-CRT). However, dose inhomogeneity may accentuate the likelihood of local recurrence, damage to nearby organs at risk (OAR), and acute and long-term adverse events^3,4^. The main acute adverse events (defined as those first observed within 90 days of the last radiotherapy session) are erythema, skin desquamation and esophagitis, while late cosmetic and functional adverse events include fibrosis of the skin, lung and deep tissues, breast and chest wall pain, skin hyper/hypopigmentation, telangiectasia, and secondary cancer^5–10^.

Intensity-modulated radiation therapy (IMRT) has been developed as means of delivering precise doses of radiation to a sometimes complex target volume. A number of clinical trials have shown that when compared with 3D-CRT, IMRT (i) provides good coverage of the target volume, (ii) reduces the delivery of high doses of radiation to OAR, and (iii) is associated with better quality of life (QoL) and lower frequencies of acute and late adverse events after conservative surgery^11–15^. In Donovan et al.’s randomized study, a change in esthetic breast appearance was less common in the IMRT arm (40%, vs. 58% in the standard arm; p = 0.008). Moreover, there was significantly less fibrosis in patients treated with IMRT^13^. Mukesh et al. reported a better dose distribution, better overall cosmetic results (OR [95% CI] = 0.68 [0.48–0.96], p = 0.027) and less frequent telangiectasia in the IMRT arm (OR [95% CI] = 0.58 [0.36–0.92], p = 0.038)^14^. Pignol et al. found an absolute reduction in exudative epithelitis (17 percentage points) in the IMRT group, relative to 2D radiotherapy (31.2% vs. 48%, respectively, p = 0.0002)^15^. However, these studies did not provide guidance how to evaluate a plan, i.e. they did not describe planning constraints. In view of these shortcomings, we recently initiated a clinical project aimed at (i) evaluating acute and medium-term toxicity in breast cancer patients treated with adjuvant IMRT, and (ii) assessing the association between adverse events and the patients’ clinical, treatment-related and dosimetric characteristics. The project was initially set up as a single-center study at the Centre Oscar Lambret cancer center (Lille, France), and the preliminary results in 114 patients have been reported^16^; we found that QoL was well maintained, and that acute esophageal toxicity was associated with a number of dosimetric factors.

We report here data collected in 288 patients treated with adjuvant breast radiotherapy in 2 centers. To the best of our knowledge, the correlation between clinical toxicity and dosimetric data has not been previously investigated.

## Patients and methods

### Study design

This was a two-center, prospective clinical study of the safety of adjuvant IMRT after breast cancer surgery. The primary objective was to describe acute adverse events. The secondary objectives were (i) identify potential prognostic factors for grade ≥ 2 acute adverse events following adjuvant IMRT, (ii) describe long-term adverse events and identify potential prognostic factors for long-term adverse events, (iii) assess QoL and esthetic outcomes, and (iv) evaluate effectiveness (in terms of time to recurrence).

### Patients and treatments

Patients were recruited at two cancer centers: the Centre Oscar Lambret (Lille, France) and the Centre Leonard de Vinci (Douai, France). The main inclusion criteria were as follows: age 18 or over, provision of informed consent, histologically proven breast cancer, and adjuvant radiotherapy after partial or total mastectomy, with or without inclusion of the axillary lymph nodes. The main exclusion criteria were metastatic disease, any severe or non-controlled disease that would have compromised participation in the study, and breast-feeding or pregnancy.

The treatment procedure was that used routinely in the investigating centers, and has been described in detail elsewhere^17^. Briefly, the clinical target volume (CTV) and the OAR were delineated according to the American Society for Radiation Oncology (ASTRO) guidelines until the end of December 2015^18^ and according to the European Society for Radiotherapy and Oncology (ESTRO) guidelines thereafter^19–21^. A 5 mm margin was added to the CTV to obtain the planning target volume (PTV). The prescribed dose for the breast, chest wall and axillary lymph nodes was 50 Gy (25 fractions of 2 Gy). This dose was delivered over 5 weeks (five irradiations/week). Patients having undergone partial mastectomy received a simultaneous integrated boost (SIB) at the surgical bed of 60 Gy, delivered in 25 fractions (25 fractions × 2.4 Gy). Treatment planning was performed in the helical mode with TomoEdge (Accuray) using a 5 cm field width. The aim was for 95% of the PTV to receive 95% of the prescribed dose. To avoid overdosing during optimization a 3 mm zone is subtracted from the outer contour—resulting in the creation of a "skin volume". The constraints for OAR are specified in Table 1.

**Table 1:** Dosimetric constraints for organs at risk.

Organ at risk	Constraint
Spinal cord	D2% < 15 Gy
Heart (left breast)	V15 < 20%
	V20 < 15%
	V25 < 10%
Ipsilateral lung	V15 < 50%
	V20 < 35%
	V30 < 20%
	V35 < 15%
Contralateral lung	V10 < 50%
	V12 < 35%
	V15 < 20%
Contralateral breast	V5 < 50%
	V7 < 35%
	V10 < 20%
	V20 < 15%

*Vx* volume receiving × dose (gray); % of the organ at risk.

### Outcomes

Adverse events were classified according to the National Cancer Institute Common Terminology Criteria for Adverse Events (version 4.0)^22^. Acute adverse events were defined as those first observed within 90 days of the last radiotherapy session. We recorded: skin toxicity (radiodermatitis, ulceration, necrosis, telangiectasia, atrophy, hyperpigmentation, and hypopigmentation), esophageal toxicity, and breast, surgical bed or scar induration. Adverse events were recorded weekly during IMRT and then 1 and 6 months and 1, 2, 3, 4 and 5 years thereafter. The purpose of the 1-month consultation was to assess early toxicity more accurately.

Health-related QoL was evaluated using the disease-specific European Organisation for Research and Treatment of Cancer (EORTC) core QoL questionnaire (QLQ-C30) and the additional breast-cancer-specific QLQ-BR-23 module, according to the EORTC manual^23,24^. The scores were linearly transformed onto a scale ranging from 0 (worst possible QoL) to 100 (best possible QoL). Aesthetic outcomes were rated by the patient and by her physician as poor, moderate, good or excellent.

### Statistical analyses

Quantitative variables were expressed as the mean ± standard deviation (SD) or the median (range), and qualitative variables were expressed as the frequency (percentage). Skin toxicity and skin fibrosis were analyzed per treated breast. Esophageal adverse events were analyzed per patient. The clinical and dosimetric variables examined for a putative association with acute or long-term adverse events and the corresponding statistical methods used have been described in detail in our previous report^16^. The sample size was calculated as follows. Given that the primary endpoint was the occurrence of grade ≥ 2 acute adverse events (considered to be treatment failures), the calculation was performed by organ (skin or esophagus). With regard to prognostic factors of toxicity, at least 10 failures per factor had to be observed. We had planned to study nine prognostic factors for skin adverse events and five for esophageal adverse events. Hence, we expected to observe at least 90 patients with a skin adverse event. According to the literature, around 30% of patients will experience a grade ≥ 2 acute skin adverse event^25^. Hence, a sample of 300 patients was required to observe 90 skin adverse events with p > 0.05 and a power of 83%.

### Ethics

The study was carried out in accordance with the precepts of the Declaration of Helsinki, approved by the local institutional review board (*Comité de Protection des Personnes Nord Ouest IV*, Lille, France; reference: SC14/03) and registered at ClinicalTrials.gov (NCT02281149). All included patients received information on the study’s objectives and procedures. In line with the French legislation on the analysis of data collected during routine care, patients gave their consent to participation.

### Consent to participate

All included individuals gave their informed consent to participation in the study and to analysis of their personal data.

## Results

### Characteristics of the patients and treatments

A total of 300 patients were included in the study, 12 of whom were subsequently excluded for various reasons: hypofractionated treatment, local progression and metastasis, withdrawal decided by the patient, withdrawal decided by the investigator, a change in treatment center, use of a different radiotherapy machine, and erroneous inclusion. Hence, 288 patients were analyzed (Table 2).

**Table 2:** Characteristics of the study population and the tumors on inclusion. Data are quoted as the number (percentage) or the median (range).

Demographic and health characteristics (n = 288)	
Age, years	55 (32–82)
**Past or current smokers**	79 (27.4%)
Number of packets per year (n = 72)	17.5 (0.5–51)
Duration (years)	28 (2–47)
**Current and/or past health conditions**	
History of heart disease	94 (23.8%)
Current diabetes	43 (14.9%)
Current dyslipidemia	91 (28.3%)
History of respiratory disease	29 (10.1%)
Family history of breast cancer	122 (42.4%)
BMI (kg/m^**2**^**)**	26.5 (16.5–48.8)
Normal weight	111 (38.8%)
Overweight (BMI ≥ 25)	95 (33.2%)
Obesity(BMI ≥ 30)	80 (28%)
**WHO score**	
0	222 (79%)
1	58 (20.6%)
2	1 (0.4%)
**Breast size**	
Small (85A-B, 90A)	31 (11.6%)
Medium (85C, 90B-C, 95A-B)	76 (28.4%)
Large (> 85C, > 90C, > 95B)	161 (60.1%)

**Tumor characteristics (n = 288)**	
**Tumor side**	
Right	130 (45.1%)
Left	142 (49.3%)
Bilateral	16 (5.6%)
**Histology**	
Invasive ductal carcinoma	217 (75.4%)
Invasive lobular carcinoma	33 (11.4%)
Other	38 (13.2%)
In situ component (n = 281)	116 (41.3%)
**SBR grade**	
SBR I	64 (24.2%)
SBR II	143 (54.2%)
SBR III	57 (21.6%)
ER + (n = 286)	243 (85.0%)
PR + (n = 286)	205 (71.7%)
HER2 + (n = 273)	42 (15.4%)
Triple-negative (n = 288)	28 (9.7%)
**pT grade (n = 265)**	
pT1	122 (46%)
pT2	110 (41.5%)
pT3	30 (11.3%)
pT4	3 (1.1%)
**pN grade (n = 275)**	
pN0	60 (21.8%)
pN1	158 (57.5%)
pN2	42 (15.3%)
pN3	15 (5.5%)

*WHO *World Health Organization, *BMI *body mass index, *SBR *Scarff-Bloom and Richardson.

One hundred and seventy patients (59%) received the SIB. Seven of the 288 patients (2.4%) did not receive the treatment not specified in the study protocol (25 × 2 Gy fractions and in some cases a SIB with 25 × 2.4 Gy fractions). All other patient received between 49.75 and 50.5 Gy in 25 fractions (breast) and (if a SIB was applied) 60 Gy in 25 fractions (surgical bed). The mean ± SD treatment time was 36.8 ± 2.0 days (median 36; range 33–45) (Table 3).

**Table 3:** Characteristics of the treatments.

Treatments	
**Surgery (n = 288)**	
Type of surgery	
Partial mastectomy	170 (59.0%)
Bilateral partial mastectomy	7 (2.4%)
Lumpectomy	3 (1.0%)
Total mastectomy	99 (34.4%)
Bilateral total mastectomy	3 (1.0%)
Total mastectomy on one side and partial mastectomy on the other	6 (2.1%)
Axillary node dissection	205 (71.2%)
Sentinel lymph node	176 (61.1%)
**Chemotherapy (n = 288)**	
Any type	209 (72.6%)
Adjuvant	159 (55.2%)
Neo-adjuvant	61 (21.2%)
**Hormone therapy (n = 283)**	
Any type	227 (80.2%)
Tamoxifen-based	90 (31.8%)
**Radiotherapy**	
Breast or chest wall PTV (n = 288)	
D50% (mean ± SD) (Gy)	49.8 ± 0.8
D95% (mean ± SD) (Gy)	47.0 ± 2.9
D2% (mean ± SD) (Gy)	56.5 ± 4.1
Concomitant boost PTV (n = 167)	
D50% (mean ± SD) (Gy)	59.4 ± 1.0
D95% (mean ± SD) (Gy)	57.2 ± 1.2
D2% (mean ± SD) (Gy)	61.2 ± 1.2
Internal mammary chain PTV (n = 258)	
D50% (mean ± SD) (Gy)	49.5 ± 1.8
D95% (mean ± SD) (Gy)	46.9 ± 3.6
D2% (mean ± SD) (Gy)	52.1 ± (1.6
Subclavicular* PTV (n = 253)	
D50% (mean ± SD) (Gy)	49.6 ± 1.3
D95% (mean ± SD) (Gy)	47.1 ± 2.1
D2% (mean ± SD) (Gy)	51.9 ± 1.1
Supraclavicular** PTV (n = 258)	
D50% (mean ± SD) (Gy)	49.9 ± 1.4
D95% (mean ± SD) (Gy)	48.2 ± 2.0
D2% (mean ± SD) (Gy)	52.0 ± 1.1

Data are quoted as the n (%) or the mean ± standard deviation (SD).

*PTV* planning target volume, *Dx%* dose received by at least x% of the volume.

*2 and 3 areas, according to ESTRO guidelines.

**Area 4, according to ESTRO guidelines.

The median follow-up time (calculated according to the reverse Kaplan–Meier method) was 2.1 years (range 6 months to 4 years). Eleven patients (3.8%) died during the follow-up period. Seven of these deaths were due to disease progression. The 2-year overall survival rate [95% confidence interval (CI)] was 97.8% [94.1–99.2%]. Seventeen cases of disease recurrence were noted (13 metastatic, 3 local + regional + metastatic, and 1 regional + metastatic). In all, 19 patients died or relapsed, giving a 2-year relapse-free survival rate [95% CI] of 93.4% [89.2–96.0%].

### Adverse events

The acute and medium-term adverse events observed during the study are summarized in Table 4. Further details of the clinical results will be presented in a subsequent publication. The most common acute adverse events were skin-related; almost all the patients experienced at least one acute skin adverse event (radiodermatitis, ulceration-necrosis, telangiectasia, atrophy, hyperpigmentation, and hypopigmentation)—primarily radiodermatitis [in 266 patients (92.4%)] and hyperpigmentation [in 178 (61.8%)]. Although the majority of these events were non-severe (i.e. no higher than grade 1), 106 patients presented with a grade ≥ 2 event (36.8% [31.2–42.7%]), and 4 presented with a grade 3 event (radiodermatitis in all 4 cases); these were the only grade 3 acute event observed in the study as a whole. The next most frequent types of acute adverse event were (mainly grade 1) esophageal damage and breast edema. Breast fibrosis and chest wall fibrosis were observed in a third of the patients, and almost all events were grade 1. The proportions of patients developing mammary fibrosis and chest wall fibrosis did not differ greatly when comparing the “total mastectomy” and “partial mastectomy” subgroups.

**Table 4:** Acute and medium-term adverse events.

Patients: n = 288	Acute	Medium-term
**Skin adverse events**	278 (96.5%)	152 (53.1%)
Grade 1	172 (59.7%)	147 (51.0%)
Grade 2	102 (35.4%)	2 (0.6%)
Grade 3	4 (1.4%)	2 (0.6%)
**Esophageal adverse events**	138 (47.9%)	6 (2.1%)
Grade 1	120 (41.7%)	6 (2.1%)
Grade 2	18 (6.3%)	-
**Edema**	57 (19.8%)	69 (24.1%)
Grade 1	55 (19.1%)	63 (22.0%)
Grade 2	1 (0.3%)	5 (1.7%)
Grade unknown	1 (0.3%)	1 (0.3%)
**Parietal fibrosis (total mastectomy, n = 120)**	36 (30.0%)	51 (42.9%)
Grade 1	34 (28.3%)	42 (35.3%)
Grade 2	2 (1.7%)	9 (7.6%)
**Breast fibrosis (partial mastectomy, n = 168)**	57 (33.9%)	68 (40.7%)
Grade 1	54 (32.1%)	59 (35.3%)
Grade 2	2 (1.2%)	9 (5.4%)
Grade unknown	1 (0.6%)	-
**Surgical scar fibrosis (total mastectomy, n = 98)**	20 (20.4% %)	52 (44.1%)
Grade 1	18 (18.4%)	45 (38.1%)
Grade 2	2 (2.0%)	7 (5.9%)
**Surgical bed fibrosis (partial mastectomy, n = 165)**	31 (18.8%)	66 (39.5%)
Grade 1	29 (17.6%)	56 (33.5%)
Grade 2	1 (0.6%)	10 (6.0%)
Grade unknown	1 (0.6%)	–

Skin adverse events were defined as radiodermatitis, ulceration-necrosis, telangiectasia, atrophy, hyperpigmentation or hypopigmentation. With regard to surgical bed fibrosis, only fibrosis absent on inclusion or of a higher grade on inclusion was considered.

With regard to the delineation method used (ASTRO: n = 88; ESTRO: n = 200), we observed several statistically significant differences in the incidence of (i) grade ≥ 1 (but not grade ≥ 2) esophageal adverse events (60.2% vs. 42.5%, respectively; p = 0.006), (ii) grade ≥ 1 (but not grade ≥ 2) breast induration in the partial mastectomy group (47.7% vs. 29.0%, respectively; p = 0.024), and (iii) grade ≥ 1 (but not grade ≥ 2) scar induration in the total mastectomy group (34.5% vs. 14.5%, respectively; p = 0.025). The mean total PTV volumes according to the ASTRO or ESTRO guidelines did not differ significantly (1052 cc ± 581 vs. 1085 cc ± 531, respectively, p = 0.65).

The most common medium-term acute adverse events at both the 13-month and 26-month time points affected the skin; at 26 months, the cumulative incidence [95% CI] was 59.2% [52.2–66.3] for grade ≥ 1 events and 1.7% [0.6–4.5] for grade ≥ 2 events. (Table 4). The incidence of fibrosis was higher in the partial mastectomy subgroup (Table 5). No respiratory toxicity was observed.

**Table 5:** Cumulative incidence of medium-term adverse events.

	Cumulative incidence [95% CI] of grade ≥ 2 adverse events after IMRT	
	At 13 months*	At 26 months*
Esophageal adverse events	–	–
Skin adverse events	1.1% [0.4–3.5]	1.7% [0.6–4.5]
Fibrosis, total mastectomy	4.5% [1.9–10.5]	7.0% [2.9–16.4]
Fibrosis, partial mastectomy	5.2% [2.6–10.2]	7.3% [3.5–15.1]
Scar fibrosis, total mastectomy	5.5% [2.5–11.9]	5.5% [2.5–11.9]
Scar fibrosis, partial mastectomy	5.7% [3.0–10.6]	7.8% [3.9–15.6]

*The cumulative incidences are quoted at 13 and 26 months (rather than 12 and 24 months) so as not to underestimate the values, since the annual check-ups often took place slightly later than 12 and 24 months. Skin adverse events were defined as radiodermatitis, ulceration-necrosis, telangiectasia, atrophy, hyperpigmentation or hypopigmentation. With regard to surgical bed fibrosis, only fibrosis absent or of a higher grade on inclusion were considered.

### Variables associated with the occurrence of grade ≥ 2 adverse events

In a univariate analysis of the whole population, age, cup size, diabetes, aesthetic score before IMRT, the type of surgery, node irradiation, SIB and the other dosimetric parameters tested were not significantly associated with the occurrence of acute adverse events. Factors positively associated (p < 0.05) with the occurrence of grade ≥ 2 acute adverse events were body mass index (BMI, as a quantitative variable), tobacco smoking, the absence of prior chemotherapy, CTV, PTV, the volume receiving 95% of the dose (V95%), boost CTV, skin volume, and the dose received by 98% of the volume (D98%). After the removal of highly correlated variables, the three variables significantly associated with the occurrence of grade ≥ 2 acute adverse events were (i) tobacco smoking (odds ratio (OR) [95% CI] = 2.10 [1.14–3.87]; p = 0.017), (ii) the absence of prior chemotherapy (0.52 [0.27–0.98]; p = 0.044) and D98% for the subclavicular skin (1.030 [1.001–1.061]; p = 0.045). Concerning breast size, an increment in CTV of 100 cc was associated with an OR [95% CI] = 1.11 (1.04–1.18) for presenting grade ≥ 2 acute skin toxicity (p = 0.003), for patients having undergone by partial mastectomy. In a quartile analysis vs. patients with a breast CTV < 610 cc, a breast CTV volume [610–811] was associated with an OR of 2.75, a CTV volume of [811–1150] was associated with an OR of 1.64, and a CTV volume >  = 1150 cc was associated with an OR of 5.96 for presenting grade ≥ 2 acute skin toxicity respectively, p = 0.002. These criteria were not selected for multivariate analysis because they were highly correlated with skin volume.

**Figure 1:**
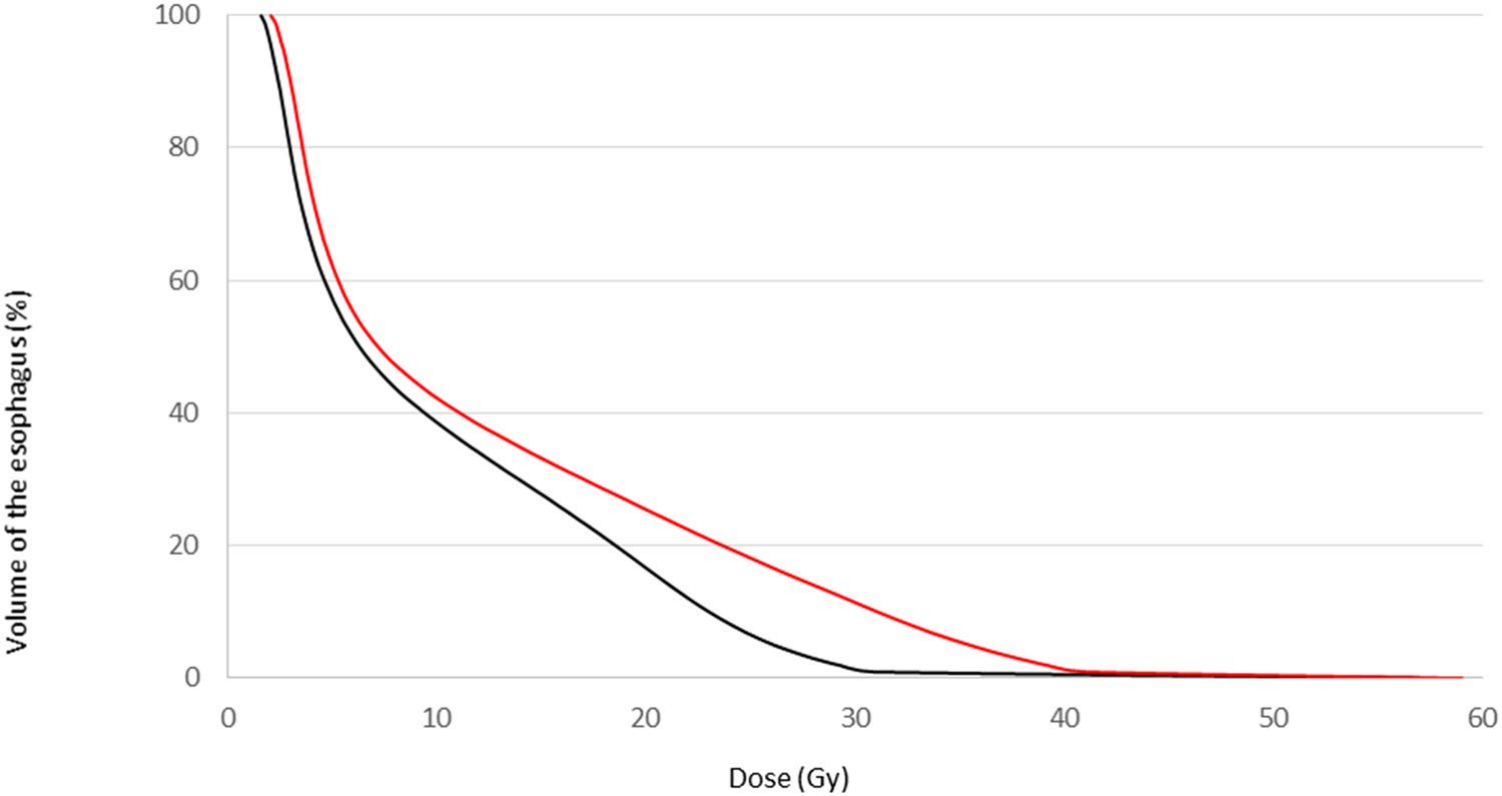
The mean dose-volume histogram for the esophagus for patients without esophageal toxicity (black line) and patients with grade ≥ 1 acute esophageal adverse events (red line).

It was not possible to search for prognostic factors for grade ≥ 2 acute esophageal adverse events, given the small number (n = 18); hence, we analyzed grade ≥ 1 events. In a univariate analysis, the following dosimetric parameters were significantly associated with grade ≥ 1 acute esophageal adverse events: the mean dose, (p < 0.0001; Fig. 1), D2% (p < 0.0001), D50% (p = 0.037), D95% (p = 0.0005), D98% (p = 0.0007), V30Gy (p < 0.0001), and V45Gy (p = 0.0001). Lymph node irradiation was the only non-dosimetric parameter significantly associated with grade ≥ 1 acute esophageal adverse events. The significantly prognostic factors identified in the univariate analysis were highly correlated; this prevented us from performing a multivariate analysis of grade ≥ 1 acute esophageal adverse events.

Given that only 4 patients experienced grade ≥ 2 medium-term skin adverse events, it was not feasible to look for associated variables; we therefore focused on grade ≥ 1 medium-term adverse events. After univariate and multivariate analyses, the following factors were found to be significant: cup size ≥ C (subhazard ratio [95% CI] 1.51 [1.03–2.22]; p = 0.035), D95% for the skin volume (1.048 [1.021–1.076]; p < 0.001), D98% for the subclavicular skin (0.920 [0.888–0.953]; p < 0.001) and low D95% for the internal mammary lymph nodes (0.978 [0.963–0.992], p = 0.003).

## Discussion

In the present prospective, two-center study of 288 patients treated for breast cancer with IMRT (including 225 (78.3%) with axillary node involvement), we found that skin adverse events were very common (observed in 96.5% of the patients) but rarely severe (with only 4 events grade 3 or higher). In a multivariate analysis, smoking and D98% for the subclavicular skin were prognostic factors for grade ≥ 2 skin adverse events, whereas prior chemotherapy was protective. Esophageal adverse events affected 47.9% of the patients but no grade ≥ 3 events were recorded. Dosimetric variables were the only prognostic factors for esophageal adverse events. The esthetic outcome for the breast 12 months post-surgery was generally good or excellent, whether judged the patients or the physicians. The patients’ QoL remained stable or improved over time following IMRT, and the overall and disease-free survival rates at 2 years were over 90%.

### Acute adverse events

As noted above, almost all the patients in the present study experienced at least one (mainly mild) acute skin adverse event. In Freedman et al.’s comparative study, the incidence of grade 2 wet desquamation was significantly lower in an IMRT cohort (21%) than in a matched 3D-CRT cohort (38%)^26^. Pignol et al.’s comparison of 2D-CRT and IMRT produced the same conclusion^15^.

In fact, most previous studies of IMRT concerned the treatment of a single breast, which limits damage to the esophagus. For bilateral treatment, Ekici et al.’s study of 14 found that 6 had an acute esophageal adverse event (all grade 1)^27^. Wang et al. studied a larger sample (n = 200) of patients having undergone IMRT after total mastectomy; only 21 patients (10.5%), experienced an acute esophageal adverse event (all but three of which were grade 1 events)^28^. Aoulad et al.’s study of 292 patients found that 58 (19.9%) experienced a grade 1 or 2 acute esophageal adverse event^29^. Caudrelier et al. reported that 37% of their patients experienced an acute esophageal adverse event (all grade 1)^30^. In comparison, our value of 47.9% is high. This might be because a high proportion of our patients displayed axillary node involvement (justifying larger treatment volumes) or because the retrospective design of previous studies possibly led to underestimation of the event frequency. Lastly, our compliance with ASTRO and ESTRO guidelines meant that the esophagus received a higher dose. In our study, we observed more esophageal toxicity using the ASTRO guidelines; this might have been due to the more cranial limit of the ASTRO target volume, relative to the ESTRO guidelines.

In the present study, 59 patients (19.8%) presented with acute breast edema (grade 1 in 57 cases). This incidence is in line with the scarce literature data. Aoulad et al. reported a value of 19.5% for grade ≥ 2 acute breast edema^29^. Harsolia et al.’s comparison of IMRT and 3D-CRT cohorts treated in the same institution found that the incidence of grade ≥ 2 acute breast edema was significantly lower in the former group (1%, vs. 29% for 3D-CRT, p = 0.02)^31^.

### Medium-term adverse events

The skin was the organ system most frequently concerned by medium-term adverse events (in 53.1% of our patients); this was primarily mild hyperpigmentation. In fact, hyperpigmentation tends to disappear with time, and so our median follow-up period of 2.1 years may have overestimated the long-term frequency of this event. The frequency of medium-term telangiectasia in our study was lower (7.0%) that the value of 31.4% was found in a study of 416 patients treated with 3D-CRT^32^. However, the results of two randomized studies were contradictory; an advantage of IMRT was reported by Mukesh et al. in the UK^14^, but not by Pignol et al. in Canada^15^. Furthermore, our study’s follow-up period was probably too short to assess truly long-term adverse events.

The esthetic outcome in the present study was judged to be “good or excellent” by 86.7% of the physicians and 84.6% of the patients. These results may be compared to the corresponding values of 96% and 88% in the Fox Chase Cancer Center study^33^. Hence, IMRT appears to be advantageous for the mid-term esthetic outcome, notably relative to 2D-CRT (64)^13,14^—probably because this measure is correlated with long-term fibrosis, edema and telangiectasia, which are generally less frequent after IMRT ^33^. However, the Canadian randomized trial failed to evidence a significant difference^34^.

In the present study, the frequency of medium-term fibrosis was 40.7% for partial mastectomy and 42.9% for total mastectomy. The corresponding frequencies of surgical scar fibrosis were 39.5% and 44.1%, respectively. Even though most of these events were grade 1, these frequencies were higher than in the literature. The Royal Marsden randomized trial of IMRT found 2-year breast fibrosis and surgical bed fibrosis rates of 16% and 37%, respectively^13^. However, the two other randomized trials did not evidence a long-term difference for IMRT^14,15^. Our study’s prospective design might have facilitated the detection of grade 1 event with little or no functional or esthetic impact. Secondly, high proportions of our patients had risk factors for the development of fibrosis (overweight, smoking, prior chemotherapy, node involvement, etc.)^35,36^. In the short term, however, the induration and fibrosis were more related to surgery than to radiotherapy. The assessment of short-term fibrosis probably increased the estimated incidence.

### Prognostic factors associated with acute adverse events

The significant variables positively associated with the occurrence of grade ≥ 2 acute adverse events were tobacco use and D98% for the subclavicular skin, whereas the prior chemotherapy was protective. In the literature, high BMI, breast volume, and smoking are confirmed risk factors for acute adverse events^29,37–41^. Prior chemotherapy is typically found to be a factor associated with toxicity too^37,42–44^. In our study patients treated intermittently with corticosteroids may have benefitted from the latter’s anti-inflammatory action. Trastuzumab has been found to be protective in some studies^39^ but not others^45^. The literature data on dosimetric parameters are far more heterogeneous. Here, the OR [95% CI] for toxicity associated with D98% for the subclavicular skin was 1.030 [1.001–1.061]; p = 0.045)*.* To the best of our knowledge, the present study is the first to have shown a correlation between skin dose and toxicity. Unfortunately, however, the dose delivered to the skin during inverse planning is not an actionable variable.

In the present study, the significant variables positively associated with grade ≥ 1 (rather than grade ≥ 2) medium-term skin adverse events in a multivariate analysis were cup size, D95% for the skin volume, D98% for the subclavicular skin and D95% for the internal mammary lymph nodes. Regardless of the technique (IMRT or 3D-CRT), women with a larger cup size are more exposed to a risk of late skin adverse events^46,47^.

### Study limitations and strengths

The study had a number of strengths. Firstly, this was one of the largest yet studies of IMRT with SIB (n = 170 patients, 59%) as an adjuvant treatment for breast cancer. Secondly, the study’s prospective design produced full, unbiased datasets on adverse events. The fact that the detected events were grade 1 suggests good treatment tolerance in the short and medium terms. Thirdly, the present study is the first to have reported on the correlation between toxicity and dosimetric factors. The study also had some limitations. Firstly, our population was relatively heterogeneous, with total mastectomy vs. breast-conserving surgery, and chemotherapy prior to IMRT, SIB, and axillary lymph node irradiation in some cases but not others. The high proportion of patients with locally advanced disease (with axillary node involvement in 78.3% of cases and prior chemotherapy in 72.6% of cases) and thus greater treatment volumes might explain the incidence of acute and long-term adverse events. We decided not to divide our study population into subgroups because this would have decreased the statistical power. Secondly, our relatively short follow-up period (median: 2.1 years) prevented us from fully assessing the incidence and nature of long-term adverse events in general and the most serious cardiac and respiratory events in particular.

## Conclusion

Adjuvant IMRT after partial or total mastectomy is associated with a low incidence of acute and medium-term adverse events. The majority of these events were non-severe, and did not degrade the cosmetic outcome for the breast. Importantly, the safety profile of IMRT is linked to dosimetric parameters (such as D2%, D50%, D95%, D98%, V30Gy and V45Gy) as well as to clinical and disease-related factors.

## Data Availability

The datasets generated during and/or analysed during the current study are available from the corresponding author on reasonable request.

## Abbreviations

2D-CRT: Two-dimensional conformational radiotherapy
3D-CRT: Three-dimensional conformational radiotherapy
AE: Adverse event
ASTRO: American Society for Radiation Oncology
BMI: Body mass index
CI: Confidence interval.
CTV: Clinical target volume
D2%: Dose received by 2% of the volume
D50%: Dose received by 50% of the volume
D95%: Dose received by 95% of the volume
D98%: Dose received by 98% of the volume
EORTC QLQ-BR23: European Organisation for Research and Treatment of Cancer Breast Cancer-Specific Quality of Life Questionnaire
EORTC QLQ-C30: European Organisation for Research and Treatment of Cancer Core Quality of Life Questionnaire
ESTRO: European Society for Radiotherapy and Oncology
IMRT: Intensity-modulated radiation therapy
OAR: Organs at risk
OR: Odds ratio
PTV: Planning target volume.
QoL: Quality of life
SBR: Scarff-Bloom and Richardson
SD: Standard deviation
SIB: Simultaneous integrated boost
V95%: Volume receiving 95% of the dose
WHO: World Health Organization
